# Dose selection for glycopyrrolate/eFlow^®^ phase III clinical studies: results from GOLDEN (Glycopyrrolate for Obstructive Lung Disease via Electronic Nebulizer) phase II dose-finding studies

**DOI:** 10.1186/s12931-017-0681-z

**Published:** 2017-12-04

**Authors:** James F. Donohue, Thomas Goodin, Robert Tosiello, Alistair Wheeler

**Affiliations:** 1 Department of Pulmonary Diseases and Critical Care Medicine, University of North Carolina School of Medicine, CB# 7020, 130 Mason Farm Road, 4th Floor Bioinformatics Building, Chapel Hill, NC 27599 USA; 2 Sunovion Pharmaceuticals Inc., Marlborough, MA USA; 3 Present address: Spyryx Biosciences, Inc., Durham, NC USA

**Keywords:** COPD, eFlow® CS, GOLDEN, Glycopyrrolate, LAMA, Nebulizer, Phase II

## Abstract

**Background:**

Long-acting muscarinic antagonists (LAMAs) are recommended for the treatment of chronic obstructive pulmonary disease (COPD). Glycopyrrolate/eFlow® is an investigational drug–device combination of the LAMA glycopyrrolate administered by an eFlow® Closed System (eFlow® CS) nebulizer. The GOLDEN 2 (NCT01706536) and GOLDEN 6 (NCT02038829) Phase II, multicenter studies were conducted to inform dose selection for the GOLDEN Phase III clinical trials. Bronchodilator responses and safety assessments supported dose selection.

**Methods:**

Subjects with moderate-to-severe COPD were randomized into 28-day parallel-group (GOLDEN 2) or 7-day crossover (GOLDEN 6) studies and received placebo, glycopyrrolate (3, 6.25, 12.5, 25, 50 or 100 μg twice daily [BID]) or aclidinium bromide 400 μg BID. The primary endpoint of both studies was change from baseline in trough forced expiratory volume in 1 s (FEV_1_). Safety assessments included the incidence of treatment-emergent adverse events (TEAEs), treatment-emergent serious adverse events, and discontinuation due to TEAE. Lung function data collected in both studies were pooled.

**Results:**

The combined GOLDEN 2 (*n* = 282) and GOLDEN 6 (*n* = 96) studies included 378 subjects. On Days 7 and 28 there were dose-ordered, statistically significant and clinically important lung function improvements in glycopyrrolate treatment groups. Specifically, on Day 7, glycopyrrolate produced >0.100 L placebo-adjusted changes from baseline in trough FEV_1_ (12.5 μg BID: 0.122 L; 25 μg BID: 0.123 L; 50 μg BID: 0.137 L) and FEV_1_ AUC_0–12_ (12.5 μg BID: 0.145 L; 25 μg BID: 0.178 L; 50 μg BID: 0.180 L). The improvements in lung function for the glycopyrrolate 25 and 50 μg BID doses were comparable to those with aclidinium bromide 400 μg BID (FEV_1_: 0.149 L; FEV_1_ AUC_0−12_: 0.172 L). Acceptable safety profiles were observed across all groups in both studies.

**Conclusions:**

The efficacy and safety findings supported selection of glycopyrrolate 25 and 50 μg BID doses for the Phase III GOLDEN studies and provided preliminary evidence for the use of nebulized glycopyrrolate as a maintenance therapy for COPD.

**Electronic supplementary material:**

The online version of this article (10.1186/s12931-017-0681-z) contains supplementary material, which is available to authorized users.

## Background

In the United States (US), approximately 12.7 million adults have a diagnosis of chronic obstructive pulmonary disease (COPD), with evidence of impaired lung function in up to 24 million Americans [1]. COPD is the third leading cause of death and is associated with substantial medical and economic disease burdens for patients and healthcare systems [2].

COPD is a heterogeneous disease requiring a spectrum of treatment options to achieve therapeutic goals [3]. Treatment response may depend on the method of delivery, drug preference and tolerability. Use of a metered dose inhaler (MDI), dry powder inhaler (DPI) or a nebulizer is appropriate for self-administered COPD therapy. MDIs and DPIs are widely prescribed, yet up to 70% of COPD patients may not receive an optimal dose due to an inability to inhale rapidly and forcefully, hold their breath after dosing, and/or exhale into the device. Over time, these issues may be associated with suboptimal outcomes [4–6].

A survey of 205 US-based pulmonologists indicated that 63% believed handheld nebulizers may be more effective than MDIs or DPIs in severe COPD, and 70% stated that nebulizers are more effective in the management of acute exacerbations [7]. Given the prevalence of COPD, several million US patients may regularly use nebulizers for COPD; however, treatment compliance can be affected by the need for frequent dosing with short-acting bronchodilators, long delivery times (≥12 min) and limited portability of jet nebulizers. In addition, standard jet nebulizers have poor delivery efficiency that may result in suboptimal drug treatment [8, 9], so there is a need for a new generation of nebulizers that can optimize treatment compliance and deliver long-acting bronchodilators into the lung.

Long-acting muscarinic antagonists (LAMAs) play a central role in the pharmacologic management of COPD [3]. Currently, there is no nebulized LAMA approved for use in COPD. Glycopyrrolate/eFlow® is a drug–device combination of glycopyrrolate administered twice daily (BID) by an investigational, innovative, vibrating membrane nebulizer (eFlow® CS; PARI Pharma GmbH, Starnberg, Germany). The eFlow® CS device delivers an acceptable respirable fraction (72% fine particle fraction) of glycopyrrolate aerosol droplets (3.7 μm mass median aerodynamic diameter, 1.7 geometric standard deviation) into the lung within 3 min with tidal breathing [10], is silent, portable, does not require patient preparation of the drug product, and is amenable to caregiver assistance. The shorter delivery time and portability may address the treatment compliance issues that have been reported with non-portable nebulizers. Nebulized glycopyrrolate delivery occurs over multiple tidal breaths, rather than during a single breath attempt, and may provide a therapeutic alternative for patients who experience difficulty operating handheld devices, exacerbate, and/or are physiologically unable to generate sufficient inspiratory pressure for inhaler use [11–14].

The Glycopyrrolate for Obstructive Lung Disease via Electronic Nebulizer (GOLDEN) 2 and GOLDEN 6 studies were conducted to inform dose selection for the GOLDEN Phase III clinical trials by characterizing the bronchodilator dose–response relationship and safety profiles of nebulized glycopyrrolate doses administered BID in subjects with moderate-to-severe COPD. Lung function data are presented pooled across both studies and individually.

## Methods

### Study design and treatment

The GOLDEN 2 study was initiated in October 2012 and completed in April 2013. GOLDEN 6 was initiated in January 2014 and completed in May 2014. The study protocols were approved by an Institutional Review Board and were conducted in accordance with the approved protocols, the International Council for Harmonization Good Clinical Practice guidelines, and the Declaration of Helsinki. All subjects provided written informed consent prior to undergoing any study procedures.

**Fig. 1:**
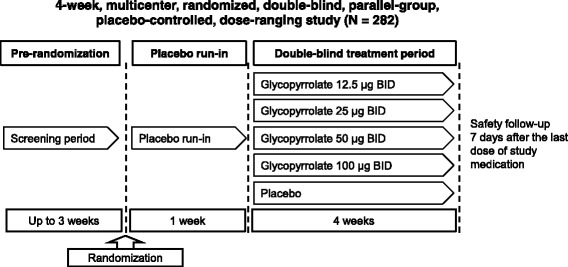
GOLDEN 2 study design. BID, twice daily

In the GOLDEN 2 study, subjects were randomized to one of five treatment groups following a 1-week placebo run-in period, and stratified by inhaled corticosteroid use and participation (yes/no) in serial spirometry assessments. The subjects received placebo, glycopyrrolate 12.5, 25, 50, or 100 μg BID for 28 days in a double-blind manner (Fig. 1).

**Fig. 2:**
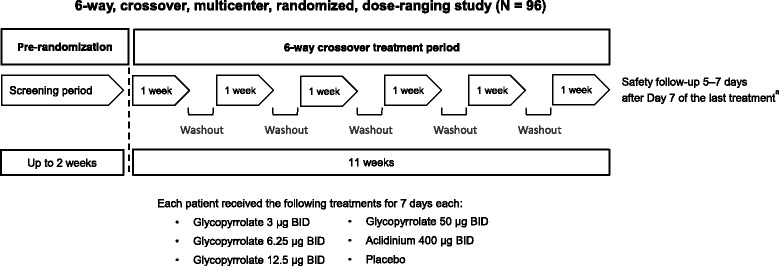
GOLDEN 6 study design. ^a^Safety follow-up was conducted 5–7 days after the Early Termination visit for subjects who discontinued the study prior to completing all scheduled treatment periods. BID, twice daily

GOLDEN 6 used a complete crossover study design, with subjects randomized to a treatment sequence consisting of six 7-day treatment periods followed by a 5- to 7-day washout (Fig. 2). During each treatment period, subjects received placebo, glycopyrrolate 3, 6.25, 12.5, or 50 μg BID, or aclidinium bromide (Tudorza® Pressair®, AstraZeneca Pharmaceuticals LP, Wilmington, DE, USA) 400 μg BID in the morning and evening. All glycopyrrolate and placebo treatments were administered double-blinded by the investigational eFlow® CS nebulizer; dosing of aclidinium bromide was open label.

### Patients

Eligible male and female subjects were 35 to 75 (GOLDEN 2) and 40 to 65 (GOLDEN 6) years old with a minimum 10 pack-year smoking history and a clinical diagnosis of moderate-to-severe COPD (GOLD 2011) [15]. Additional inclusion criteria were a forced expiratory volume in 1 s (FEV_1_) between 30% and 70% (GOLDEN 2) and 40% and 70% (GOLDEN 6) of the predicted normal, an FEV_1_/forced vital capacity ratio <0.70 and demonstrated reversibility (FEV_1_ ≥12% and 0.100 L) following post-bronchodilator (inhalation of ipratropium bromide) spirometry at screening. Subjects with current or history of unstable cardiac and/or respiratory disease (including asthma) or unstable comorbidities were excluded. Other exclusion criteria included systemic steroid therapy, respiratory infection, and a COPD exacerbation requiring hospitalization or need for increased treatments for COPD within 1.5 to 3 months of screening. Subjects using oxygen therapy for >10 h daily were also excluded.

### Study objectives and endpoints

The primary objective of both studies was to confirm the efficacy and dose–response relationship of glycopyrrolate BID in subjects with moderate-to-severe COPD, and identify doses for the GOLDEN Phase III clinical trials. Safety and tolerability of glycopyrrolate/eFlow® were secondary objectives.

The GOLDEN 2 primary efficacy endpoint was change from baseline in morning trough FEV_1_ on Day 28. Secondary endpoints included standardized change from baseline in both FEV_1_ area under the curve from 0 to 12 h (AUC_0–12_) and peak FEV_1_ on Day 28. Other endpoints included change from baseline in FEV_1_ AUC_0–12_ and trough FEV_1_ on Day 7.

The primary and secondary efficacy endpoints for GOLDEN 6 were placebo-adjusted change from baseline in morning trough FEV_1_ and standardized change from baseline in FEV_1_ AUC_0−12_, both on Day 7.

Safety assessments included the incidence of treatment-emergent adverse events (TEAEs), serious TEAEs, discontinuations due to TEAEs, changes in clinical laboratory assessments, vital signs and electrocardiograms (ECG, including QT interval), and physical examination findings. Vital signs, clinical laboratory assessments, and ECG were collected at Days 1, 7 and 28 for GOLDEN 2, and at Days 1 and 7 for GOLDEN 6.

### Statistical analyses

For GOLDEN 2, 45 subjects per treatment arm were required to provide approximately 80% power to detect a treatment difference of 0.12 L in the primary efficacy endpoint (mean change in trough FEV_1_) between each glycopyrrolate dose group and placebo (at a significance level of 0.05, assuming a standard deviation [SD] of 0.200 L and using a two-sided test). For GOLDEN 6, 66 subjects were required to provide approximately 90% power to detect a treatment difference of 0.10 L in the primary efficacy endpoint (mean change in trough FEV_1_) between each glycopyrrolate dose group and placebo (at a significance level of 0.0125, assuming an SD of 0.176 L and using a two-sided test). Additional subjects (total *n* = 78) were required in GOLDEN 6 for the trial to have sufficient power for the key secondary endpoint, FEV_1_ AUC_0–12_. Accounting for potential dropouts, the planned enrollment for GOLDEN 2 was 275 subjects and 96 subjects for GOLDEN 6.

All subjects who were randomized and received at least one dose of study drug were included in the statistical analyses of baseline characteristics, efficacy, and safety. Missing data were treated as missing at random. Sensitivity analyses were performed to assess the impact of missing data.

Each study was analyzed separately. Subsequently, the data were pooled to increase the sample size in the overlapping glycopyrrolate dose groups (12.5 and 50 μg BID) and fully characterize the dose–response profile. Pooling of these Phase II data was justified based on the overlap of doses, BID dose regimen, blinding of doses, nebulizer device, time points, similarity of study populations, and primary endpoint (trough FEV_1_).

Trough FEV_1_ was calculated as the mean of two FEV_1_ values obtained between 23 and 24 h after the morning dose of study drug on Day 7 (GOLDEN 6) or Day 28 (GOLDEN 2). Change in trough FEV_1_ was calculated as trough FEV_1_ minus baseline FEV_1_ (the mean of two FEV_1_ values obtained at 45 and 15 min prior to the morning dose on Day 1).

Least squares (LS) means and 95% confidence intervals (CIs) for the pooled study data were derived from an analysis of covariance (ANCOVA) model with change from baseline in trough FEV_1_ or FEV_1_ AUC_0–12_ as the response variable, a factor for treatment group, and with baseline FEV_1_ as a covariate.

Safety parameters were analyzed descriptively for the safety population. All adverse events (AEs) were classified using the Medical Dictionary for Regulatory Activities Version 15.1.

All statistical procedures were performed using SAS® Version 9.2 (SAS Institute Inc., Cary, NC, USA).

## Results

### Patient demographics and baseline characteristics

A total of 282 subjects were randomized in GOLDEN 2, and 96 were randomized in GOLDEN 6. In the pooled population of 378 subjects, 27 (7%) discontinued; the most frequent reason was AEs (14 [5%] in GOLDEN 2 and 5 [5.2%] in GOLDEN 6).

Pooled patient demographics and baseline characteristics are presented in Table 1 (data for the individual studies are available in Additional file 1: Tables S1 and S2). Mean age was 59 years (range: 40–75 years). Most subjects were white (90%) and 52% were female. The proportion of subjects with severe COPD ranged from 37.2% to 51.9%. The majority of subjects (60.3%) were current smokers and the mean duration of smoking history ranged from 50.1 to 54.0 pack-years.

**Table 1:** Patient demographics and baseline characteristics (pooled population)

Parameter	Placebo	Glycopyrrolate						Aclidinium	Total
	(*n* = 149)	3 μg BID (*n* = 91)	6.25 μg BID (*n* = 92)	12.5 μg BID (*n* = 145)	25 μg BID (*n* = 54)	50 μg BID (*n* = 149)	100 μg BID (*n* = 59)	400 μg BID (*n* = 94)	(*N* = 378)
Mean (SD) age, years	57.7 (7.65)	54.5 (5.90)	54.4 (5.90)	56.9 (7.37)	59.6 (8.98)	56.4 (7.49)	59.4 (7.65)	54.6 (5.92)	59.0 (8.05)
Age <65 years, *n* (%)	120 (80.5)	89 (97.8)	90 (97.8)	124 (85.5)	40 (74.1)	126 (84.6)	42 (71.2)	92 (97.9)	278 (73.5)
Gender, *n* (%)									
Female	80 (53.7)	49 (53.8)	49 (53.3)	76 (52.4)	30 (55.6)	73 (49.0)	34 (57.6)	50 (53.2)	197 (52.1)
Male	69 (46.3)	42 (46.2)	43 (46.7)	69 (47.6)	24 (44.4)	76 (51.0)	25 (42.4)	44 (46.8)	181 (47.9)
Race, *n* (%)									
White	131 (87.9)	81 (89.0)	83 (90.2)	132 (91.0)	51 (94.4)	135 (90.6)	49 (83.1)	84 (89.4)	339 (89.7)
Black/African American	17 (11.4)	10 (11.0)	9 (9.8)	13 (9.0)	3 (5.6)	13 (8.7)	10 (16.9)	10 (10.6)	37 (9.8)
American Indian/Alaskan Native	1 (0.7)	0	0	0	0	1 (0.7)	0	0	2 (0.5)
Post-bronchodilator FEV_1_, *n* (%)									
<50% predicted	60 (40.3)	34 (37.4)	34 (37.0)	59 (40.7)	28 (51.9)	62 (41.6)	25 (42.4)	35 (37.2)	NA
≥50% predicted	89 (59.7)	57 (62.6)	58 (63.0)	86 (59.3)	26 (48.1)	87 (58.4)	34 (57.6)	59 (62.8)	NA

*BID* twice daily, *FEV*_*1*_ forced expiratory volume in 1 s, *NA* not available, *SD* standard deviation

Treatment compliance during the double-blind treatment period was 95.8–97.2% across treatment groups in GOLDEN 2, and 98.9–99.3% in GOLDEN 6.

### Efficacy

In the pooled data, nebulized glycopyrrolate showed significant improvements in change from baseline in trough FEV_1_ and FEV_1_ AUC_0−12_ on Day 7 (Tables 2 and 3). Individual study data for the glycopyrrolate doses of 12.5 and 50 μg BID are reported below and for the remaining treatment groups in Additional file 1: Tables S3–S7.

**Table 2:** Change from baseline in trough FEV_1_ on Day 7 (pooled population)

Parameter	Placebo	Glycopyrrolate						Aclidinium
	(*n* = 149)	3 μg BID (*n* = 91)	6.25 μg BID (*n* = 92)	12.5 μg BID (*n* = 145)	25 μg BID (*n* = 54)	50 μg BID (*n* = 149)	100 μg BID (*n* = 59)	400 μg BID (*n* = 94)
Baseline FEV_1_								
*n*	149	91	92	144	54	149	59	94
Mean (SD), L	1.296 (0.429)	1.363 (0.429)	1.380 (0.440)	1.302 (0.421)	1.205 (0.425)	1.321 (0.433)	1.202 (0.463)	1.395 (0.464)
FEV_1_ on Day 7								
*n*	139	86	88	137	51	139	57	86
Mean (SD), L	1.304 (0.419)	1.375 (0.422)	1.454 (0.482)	1.396 (0.435)	1.313 (0.434)	1.436 (0.440)	1.359 (0.491)	1.508 (0.434)
Change from baseline in FEV_1_ on Day 7								
Mean (SD), L	−0.024 (0.214)	−0.012 (0.186)	0.063 (0.200)	0.100 (0.193)	0.108 (0.202)	0.113 (0.214)	0.154 (0.170)	0.120 (0.187)
LS mean (SE), L	−0.024 (0.017)	−0.007 (0.021)	0.068 (0.021)	0.097 (0.017)	0.099 (0.028)	0.113 (0.017)	0.145 (0.026)	0.125 (0.021)
95% CI	−0.057, 0.009	−0.049, 0.034	0.026, 0.109	0.064, 0.131	0.045, 0.153	0.081, 0.146	0.094, 0.196	0.083, 0.167
Placebo-adjusted change from baseline in FEV_1_ on Day 7								
LS mean (SE), L	–	0.017 (0.027)	0.092 (0.027)	0.122 (0.024)	0.123 (0.032)	0.137 (0.024)	0.169 (0.031)	0.149 (0.027)
95% CI	–	−0.036, 0.070	0.039, 0.144	0.075, 0.168	0.060, 0.186	0.091, 0.184	0.108, 0.230	0.096, 0.202

*BID* twice daily, *CI* confidence interval, *FEV*_*1*_ forced expiratory volume in 1 s, *LS* least squares, *SD* standard deviation, *SE* standard error

**Table 3:** Standardized change from baseline in FEV_1_ AUC_0–12_ on Day 7 (pooled population)

Parameter	Placebo	Glycopyrrolate						Aclidinium
	(*n* = 149)	3 μg BID (*n* = 91)	6.25 μg BID (*n* = 92)	12.5 μg BID (*n* = 145)	25 μg BID (*n* = 54)	50 μg BID (*n* = 149)	100 μg BID (*n* = 59)	400 μg BID (*n* = 94)
Baseline FEV_1_								
*n*	149	91	92	144	54	149	59	94
Mean (SD), L	1.296 (0.429)	1.363 (0.429)	1.380 (0.440)	1.302 (0.421)	1.205 (0.425)	1.321 (0.433)	1.202 (0.463)	1.395 (0.464)
Standardized change from baseline in FEV_1_ AUC_0–12_ on Day 7								
*n*	146	91	92	143	53	146	59	90
Mean (SD), L	−0.016 (0.187)	0.034 (0.178)	0.068 (0.181)	0.130 (0.174)	0.167 (0.200)	0.163 (0.201)	0.210 (0.143)	0.153 (0.224)
LS mean (SE), L	−0.016 (0.016)	0.036 (0.020)	0.071 (0.020)	0.129 (0.016)	0.162 (0.026)	0.163 (0.016)	0.205 (0.025)	0.156 (0.020)
95% CI	−0.047, 0.014	−0.003, 0.074	0.032, 0.109	0.098, 0.160	0.112, 0.213	0.133, 0.194	0.157, 0.253	0.117, 0.195
Placebo-adjusted change from baseline in FEV_1_ AUC_0–12_ on Day 7								
LS mean (SE), L	–	0.052 (0.025)	0.087 (0.025)	0.145 (0.022)	0.178 (0.030)	0.180 (0.022)	0.221 (0.029)	0.172 (0.025)
95% CI	–	0.003, 0.101	0.038, 0.136	0.102, 0.188	0.120, 0.237	0.137, 0.223	0.164, 0.278	0.123, 0.221

*AUC* area under the curve, *BID* twice daily, *CI* confidence interval, *FEV*_*1*_ forced expiratory volume in 1 s, *LS* least squares, *SD* standard deviation, *SE* standard error

#### Change from baseline in trough FEV_1_

Glycopyrrolate 12.5 and 50 μg BID showed significant improvement in LS mean placebo-adjusted change from baseline in trough FEV_1_ on Day 7 and Day 28 in GOLDEN 2, and on Day 7 in GOLDEN 6 (GOLDEN 2, Day 7: 0.118 and 0.149 L; GOLDEN 2, Day 28: 0.117 and 0.146 L; GOLDEN 6, Day 7: 0.109 and 0.138 L, respectively) (Additional file 1: Tables S3 and S4). Change from baseline in FEV_1_ over 24 h on Day 28 (GOLDEN 2) and Day 7 (GOLDEN 6), for all doses, is presented in Additional file 1: Figs. S1 and S2.

**Fig. 3:**
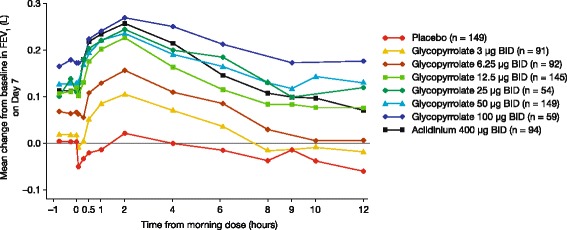
Mean change from baseline in FEV_1_ over time on Day 7 (pooled population). BID, twice daily. FEV_1_, forced expiratory volume in 1 second

For the pooled data, the change from baseline in trough FEV_1_ on Day 7 was significantly greater for all doses of nebulized glycopyrrolate (except the glycopyrrolate 3 μg BID) than for placebo (Table 2, Fig. 3). The placebo-adjusted LS mean change from baseline on Day 7 showed dose-related increases in trough FEV_1_ of 0.122, 0.123, 0.137, and 0.169 L for the glycopyrrolate 12.5, 25, 50, and 100 μg BID doses respectively. Placebo-adjusted changes from baseline in trough FEV_1_ of subjects who received glycopyrrolate 50 μg BID showed similar increases to those of subjects who received aclidinium bromide (0.137 and 0.149 L, respectively).

#### Change from baseline in FEV_1_ AUC_0–12_

**Fig. 4:**
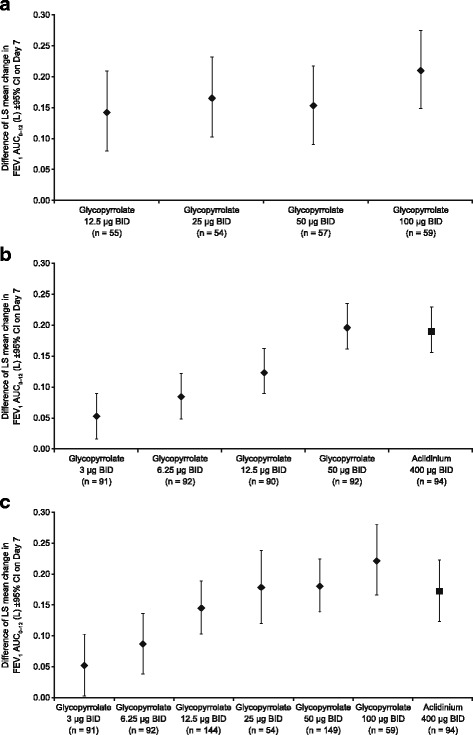
Placebo-adjusted change from baseline in FEV_1_ AUC_0-12_ on Day 7 (a: GOLDEN 2; b: GOLDEN 6; c: pooled population). AUC, area under the curve. BID, twice daily. CI, confidence interval. FEV_1_, forced expiratory volume in 1 second. LS, least squares

In GOLDEN 2, on Day 7 (Fig. 4a) and Day 28, both glycopyrrolate 12.5 and 50 μg BID doses produced significant increases in LS mean placebo-adjusted change from baseline in FEV_1_ AUC_0–12_ (Day 7: 0.143 and 0.153 L; Day 28: 0.136 and 0.105 L, respectively; Additional file 1: Table S5).

Increases in LS mean placebo-adjusted change from baseline in FEV_1_ AUC_0–12_ on Day 7 (Fig. 4b) in GOLDEN 6 were observed for both glycopyrrolate 12.5 μg BID (0.126 L) and 50 μg BID (0.196 L), with the improvement for glycopyrrolate 50 μg BID being comparable to that with aclidinium 400 μg BID (0.190 L) (Additional file 1: Table S6).

In the pooled data, on Day 7, a dose-related improvement was apparent for glycopyrrolate, in terms of the placebo-adjusted standardized change from baseline in FEV_1_ AUC_0–12_ (Table 3; Fig. 4c). Compared with the placebo-adjusted change in FEV_1_ AUC_0–12_ versus baseline with aclidinium 400 μg BID (0.172 L), there was less improvement with glycopyrrolate 12.5 μg BID (0.145 L), and more improvement with glycopyrrolate 25 μg BID (0.178 L) and glycopyrrolate 50 μg BID (0.180 L).

#### Change from baseline in peak FEV_1_ (GOLDEN 2 only)

At Day 28, in GOLDEN 2, improvements were seen in placebo-adjusted change from baseline in peak FEV_1_ for all glycopyrrolate doses including 12.5 and 50 μg BID (0.168 and 0.165 L, respectively) (Additional file 1: Table S7).

#### Characterization of the dose–response relationship for glycopyrrolate/eFlow®

Improvements in placebo-adjusted trough FEV_1_ with nebulized glycopyrrolate were clinically meaningful within the dose range of 12.5 to 100 μg BID. Although the group mean changes with glycopyrrolate 12.5 μg BID were >0.100 L, the lower bound of the 90% CI was <0.100 L. In addition, a statistical comparison between the 12.5 μg and 50 μg groups in GOLDEN 6 showed that the 50 μg BID dose was statistically superior, as measured by change from baseline in FEV_1_ AUC_0–12_. Exploratory model-fitting indicated that a sigmoidal model was the best fit to the trough FEV_1_ data, and showed that glycopyrrolate doses of 3 to 50 μg were situated on the monotonically increasing dose–response curve.

### Safety

#### Treatment-emergent adverse events

Overall, TEAEs were reported for 87 subjects in GOLDEN 2, and for 62 subjects in GOLDEN 6. In GOLDEN 2, the total incidence of TEAEs was comparable between the placebo group (26%) and the glycopyrrolate groups (12.5 μg BID, 35%; 25 μg BID, 33%; 50 μg BID, 32%; 100 μg BID, 29%; Table 4). Similar TEAE incidences were observed in the treatment groups in GOLDEN 6 (glycopyrrolate 3 μg BID, 24%; 6.25 μg BID, 25%; 12.5 μg BID, 27%; 50 μg BID, 15%; aclidinium 400 μg BID, 26%), with an incidence of 12% in the placebo group. The most frequent TEAEs seen with glycopyrrolate were COPD exacerbation (1.7–7.4%; placebo 1.8%) and headache (0–5.1%; placebo 1.8%) in GOLDEN 2, and hypertension (1.1–4.4%; placebo 0%) and cough (3.3–6.7%; placebo 2.2%) in GOLDEN 6.

**Table 4:** Summary of treatment-emergent adverse events

	GOLDEN 2					GOLDEN 6					
	Placebo	Glycopyrrolate				Placebo	Glycopyrrolate				Aclidinium
	(*n* = 57)	12.5 μg BID (*n* = 55)	25 μg BID (*n* = 54)	50 μg BID (*n* = 57)	100 μg BID (*n* = 59)	(*n* = 92)	3 μg BID (*n* = 91)	6.25 μg BID (*n* = 92)	12.5 μg BID (*n* = 90)	50 μg BID (*n* = 92)	400 μg BID (*n* = 94)
Any TEAE, *n* (%)	15 (26.3)	19 (34.5)	18 (33.3)	18 (31.6)	17 (28.8)	11 (12.0)	22 (24.2)	23 (25.0)	24 (26.7)	14 (15.2)	24 (25.5)
Potentially related TEAE^a^, *n* (%)	0	5 (9.1)	2 (3.7)	2 (3.5)	4 (6.8)	4 (4.3)	5 (5.5)	5 (5.4)	9 (10.0)	7 (7.6)	11 (11.7)
Serious TEAE, *n* (%)	2 (3.5)	2 (3.6)	2 (3.7)	1 (1.8)	3 (5.1)	1 (1.1)	0	2 (2.2)	1 (1.1)	0	3 (3.2)
Discontinuations due to TEAE, *n* (%)	2 (3.5)	3 (5.5)	4 (7.4)	3 (5.3)	1 (1.7)	1 (1.1)	0	0	1 (1.1)	0	2 (2.1)

*BID* twice daily, *TEAE* treatment-emergent adverse event

^a^Considered by the Investigator to have a definite, probable, or possible relationship to study drug

Neither study showed evidence of a dose-related relationship in terms of the incidence of any specific TEAE. In subjects treated with aclidinium 400 μg BID, the most common TEAEs were dysgeusia (8.5%), and bronchitis, cough, and nausea (all 2.1%).

Discontinuations due to TEAEs are presented in Table 4.

#### Serious treatment-emergent adverse events and deaths

The overall incidence of serious TEAEs reported for glycopyrrolate/eFlow® was comparable to that for placebo and aclidinium (GOLDEN 2: placebo, 3.5%; glycopyrrolate 12.5 μg BID, 3.6%; 25 μg BID, 3.7%; 50 μg BID, 1.8%; 100 μg BID, 5.1%. GOLDEN 6: placebo, 1.1%; glycopyrrolate 3 μg BID, 0%; 6.25 μg BID, 2.2%; 12.5 μg BID, 1.1%; 50 μg BID, 0%; aclidinium 400 μg BID, 3.2%; Table 4). The only serious TEAE to occur in more than one subject receiving glycopyrrolate was COPD exacerbation, which occurred in two subjects in GOLDEN 2 (one in the 25 μg BID and one in the 100 μg BID dose groups) and two subjects in GOLDEN 6 (one in the 6.25 μg BID and one in the 12.5 μg BID dose groups).

Two subjects died, one in GOLDEN 2 (a female subject who had severe cardiac arrest prior to receiving double-blind medication) and one in GOLDEN 6 (a male with presumed poly-drug [i.e. opiate] toxicity 2 days after the last dose of glycopyrrolate 6.25 μg BID, which was not considered to be treatment related).

#### Vital signs, clinical laboratory, and electrocardiogram parameters

In both studies, no clinically meaningful findings or trends were noted in vital signs, clinical laboratory assessments, or ECG (including QTc-F) parameters between treatment groups (data not shown).

## Discussion

This analysis of pooled data from two Phase II randomized, placebo- and/or active-controlled studies was conducted to characterize the dose–response relationship for nebulized glycopyrrolate, and inform dose selection for the Phase III studies. In subjects with moderate-to-severe COPD, treatment with glycopyrrolate/eFlow® was associated with significant improvements in pulmonary function versus placebo, and was generally well tolerated.

Analysis of the pooled population suggests a sigmoidal dose–response relationship. Glycopyrrolate 3 μg BID was deemed the ‘no effect’ dose, with doses up to 50 μg BID situated on the monotonically increasing curve, indicating increased response with increasing dose. The glycopyrrolate 6.25 μg BID and 12.5 μg BID doses had statistically significant but clinically suboptimal effects. At doses of 25 and 50 μg BID, clinically meaningful improvements in the change from baseline in trough FEV_1_ and change from baseline in FEV_1_ AUC_0–12_ were observed, compared with placebo, with greater improvements seen with 50 μg BID than with 25 μg BID. These improvements with glycopyrrolate/eFlow® were either comparable or superior to those observed with aclidinium 400 μg BID. Based on these data, glycopyrrolate doses of 25 μg BID and 50 μg BID were selected for further evaluation. Pooled and individual study data supported the dose selection.

Although previous studies with glycopyrrolate/eFlow® administered once daily (QD) showed statistically significant increases in trough FEV_1_ [16, 17], a substantial incremental improvement was seen with BID dosing. Glycopyrrolate 50 μg QD administered for 7 days improved the trough FEV_1_ by 0.068 L, while the pooled improvement in trough FEV_1_ with glycopyrrolate 50 μg BID for 7 days was 0.137 L in these studies.

## Conclusion

In the Phase II GOLDEN 2 and 6 studies, the statistical and clinical improvements in lung function, relative to placebo on Days 7 and 28, and acceptable safety profile support the selection of the glycopyrrolate 25 and 50 μg BID doses for the GOLDEN Phase III program. The combined study results provide preliminary evidence for the use of nebulized glycopyrrolate BID as a maintenance therapy for the treatment of COPD. Nebulized glycopyrrolate may provide patients and physicians with an additional treatment option for moderate-to-severe COPD, and provide a therapeutic alternative for patients who experience physical and/or physiological difficulty using handheld inhalers.

## Additional file

Additional file 1 Online Supplementary Data. **Table S1.** Patient demographics and baseline characteristics, GOLDEN 2 (ITT population). **Table S2.** Patient demographics and baseline characteristics, GOLDEN 6 (safety population). **Table S3.** Change from baseline in trough FEV_1_ on Day 7 and Day 28, GOLDEN 2 (ITT population). **Table S4.** Change from baseline in trough FEV_1_ on Day 7, GOLDEN 6 (efficacy population). **Table S5.** Standardized change from baseline in FEV_1_AUC_0–12_ on Day 7 and Day 28, GOLDEN 2 (ITT population). **Table S6.** Standardized change from baseline in FEV_1_ AUC_0–12_ on Day 7, GOLDEN 6 (efficacy population). **Table S7.** Change from baseline in peak FEV_1_ on Day 28, GOLDEN 2 (ITT population). **Figure S1.** Least squares mean change from baseline in FEV_1_ over time on Day 28 (GOLDEN 2 Substudya ITT population). **Figure S2.** Mean change from baseline in FEV_1_ over time on Day 7 (GOLDEN 6 efficacy population). (DOCX 269 kb)

## Abbreviations

AE: Adverse event
ANCOVA: Analysis of covariance
AUC: Area under the curve
BID: Twice daily
CI: Confidence interval
COPD: Chronic obstructive pulmonary disease
DPI: Dry powder inhaler
ECG: Electrocardiogram
FEV_1_: Forced expiratory volume in one second
GOLDEN: Glycopyrrolate for Obstructive Lung Disease via Electronic Nebulizer
LAMA: Long-acting muscarinic antagonist
LS: Least squares
MDI: Metered dose inhaler
QD: Once daily
QTc-F: Corrected QT (Fridericia’s formula)
SD: Standard deviation
SE: Standard error
TEAE: Treatment-emergent adverse event
US: United States
