# Supplementary material for: Dose selection for glycopyrrolate/eFlow® phase III clinical studies: results from GOLDEN (Glycopyrrolate for Obstructive Lung Disease via Electronic Nebulizer) phase II dose-finding studies
Source: Respir Res. 2017 Dec 4;18:202. doi: 10.1186/s12931-017-0681-z (PMC5715551; doi:10.1186/s12931-017-0681-z)
Supplement: Additional file 1 — Online Supplementary Data. Table S1. Patient demographics and baseline characteristics, GOLDEN 2 (ITT population). Table S2. Patient demographics and baseline characteristics, GOLDEN 6 (safety population). Table S3. Change from baseline in trough FEV1 on Day 7 and Day 28, GOLDEN 2 (ITT population). Table S4. Change from baseline in trough FEV1 on Day 7, GOLDEN 6 (efficacy population). Table S5. Standardized change from baseline in FEV1AUC0–12 on Day 7 and Day 28, GOLDEN 2 (ITT population). Table S6. Standardized change from baseline in FEV1 AUC0–12 on Day 7, GOLDEN 6 (efficacy population). Table S7. Change from baseline in peak FEV1 on Day 28, GOLDEN 2 (ITT population). Figure S1. Least squares mean change from baseline in FEV1 over time on Day 28 (GOLDEN 2 Substudya ITT population). Figure S2. Mean change from baseline in FEV1 over time on Day 7 (GOLDEN 6 efficacy population). (DOCX 269 kb) [file 12931_2017_681_MOESM1_ESM.docx]

**Online Supplementary Data**

*Dose selection for glycopyrrolate/eFlow® Phase III clinical studies: results from GOLDEN (Glycopyrrolate for Obstructive Lung Disease via Electronic Nebulizer) Phase II dose-finding studies*

James F. Donohue, Thomas Goodin, Robert Tosiello, Alistair Wheeler

**Table S1** Patient demographics and baseline characteristics, GOLDEN 2 (ITT population)

| **Parameter** | **Placebo** | **Glycopyrrolate** | | | | **Total** |
| --- | --- | --- | --- | --- | --- | --- |
|  | **(*n* = 57)** | **12.5 µg BID**  **(*n* = 55)** | **25 µg BID**  **(*n* = 54)** | **50 µg BID**  **(*n* = 57)** | **100 µg BID**  **(*n* = 59)** | **(*N* = 282)** |
| **Mean (SD) age, years** | 63.0 (7.3) | 60.9 (7.8) | 59.6 (9.0) | 59.4 (8.7) | 59.4 (7.7) | 60.5 (8.2) |
| **Age <65 years, *n* (%)** | 30 (52.6) | 36 (65.5) | 40 (74.1) | 36 (63.2) | 42 (71.2) | 184 (65.2) |
| **Gender, *n* (%)** | | | | | | |
| Female | 31 (54.4) | 28 (50.9) | 30 (55.6) | 24 (42.1) | 34 (57.6) | 147 (52.1) |
| Male | 26 (45.6) | 27 (49.1) | 24 (44.4) | 33 (57.9) | 25 (42.4) | 135 (47.9) |
| **Race, *n* (%)** | | | | | | |
| White | 49 (86.0) | 51 (92.7) | 50 (92.6) | 53 (93.0) | 49 (83.1) | 252 (89.4) |
| Black/African American | 7 (12.3) | 4 (7.3) | 3 (5.6) | 3 (5.3) | 10 (16.9) | 27 (9.6) |
| American Indian/ Alaskan Native | 1 (1.8) | 0 | 0 | 1 (1.8) | 0 | 2 (0.7) |
| Other (Mexican American) | 0 | 0 | 1 (1.9) | 0 | 0 | 1 (0.4) |
| **Post-bronchodilator FEV_1_, *n* (%)** | | | | | | |
| <50% predicted | 26 (45.6) | 26 (47.3) | 28 (51.9) | 28 (49.1) | 25 (42.4) | 133 (47.2) |
| ≥50% predicted | 31 (54.4) | 29 (52.7) | 26 (48.1) | 29 (50.9) | 34 (57.6) | 149 (52.8) |

BID, twice daily. FEV_1_, forced expiratory volume in 1 second. ITT, intent-to-treat. SD, standard deviation.

**Table S2** Patient demographics and baseline characteristics, GOLDEN 6 (safety population)

| **Parameter** | **All subjects**  **(*N*= 96)** |
| --- | --- |
| **Mean (SD) age, years** | 54.6 (5.9) |
| **Age <65 years, *n* (%)** | 94 (97.9) |
| **Gender, *n* (%)** | |
| Female | 50 (52.1) |
| Male | 46 (47.9) |
| **Race, *n* (%)** | |
| White | 86 (89.6) |
| Black/African American | 10 (10.4) |
| **Post-bronchodilator FEV_1_, *n* (%)** | |
| <50% predicted | 35 (36.5) |
| ≥50% predicted | 61 (63.5) |

FEV_1_, forced expiratory volume in 1 second. SD, standard deviation.

**Table S3** Change from baseline in trough FEV_1_ on Day 7 and Day 28, GOLDEN 2 (ITT population)

| **Parameter** | **Placebo** | **Glycopyrrolate** | | | |
| --- | --- | --- | --- | --- | --- |
|  | **(*n* = 57)** | **12.5 µg BID**  **(*n* = 55)** | **25 µg BID**  **(*n* = 54)** | **50 µg BID**  **(*n* = 57)** | **100 µg BID**  **(*n* = 59)** |
| **Baseline FEV_1_** | | | | | |
| *n* | 57 | 54 | 54 | 57 | 59 |
| Mean (SD), L | 1.169 (0.403) | 1.227 (0.434) | 1.205 (0.425) | 1.243 (0.449) | 1.202 (0.463) |
| **Change from baseline in FEV_1_ on Day 7** | | | | | |
| *n* | 53 | 51 | 51 | 53 | 57 |
| Mean (SD), L | –0.009 (0.138) | 0.104 (0.162) | 0.108 (0.202) | 0.133 (0.205) | 0.154 (0.170) |
| LS mean (SE), L | –0.024 (0.023) | 0.094 (0.023) | 0.096 (0.023) | 0.125 (0.023) | 0.147 (0.022) |
| 95% CI | –0.069, 0.022 | 0.048, 0.140 | 0.050, 0.142 | 0.080, 0.170 | 0.103, 0.190 |
| **Placebo-adjusted change from baseline in FEV_1_ on Day 7** | | | | | |
| LS mean (SE), L | - | 0.118 (0.032) | 0.119 (0.032) | 0.149 (0.032) | 0.171 (0.032) |
| 95% CI | - | 0.054, 0.182 | 0.055, 0.183 | 0.086, 0.212 | 0.108, 0.233 |
| **Change from baseline in FEV_1_ on Day 28** | | | | | |
| *n* | 51 | 50 | 48 | 50 | 56 |
| Mean (SD), L | 0.000 (0.167) | 0.106 (0.180) | 0.133 (0.265) | 0.134 (0.238) | 0.179 (0.217) |
| LS mean (SE), L | –0.011 (0.029) | 0.106 (0.029) | 0.117 (0.029) | 0.135 (0.029) | 0.166 (0.028) |
| 95% CI | –0.068, 0.046 | 0.049, 0.163 | 0.059, 0.175 | 0.079, 0.191 | 0.112, 0.220 |
| **Placebo-adjusted change from baseline in FEV_1_ on Day 28** | | | | | |
| LS mean (SE), L | - | 0.117 (0.041) | 0.128 (0.041) | 0.146 (0.040) | 0.177 (0.040) |
| 95% CI | - | 0.037, 0.197 | 0.048, 0.209 | 0.067, 0.226 | 0.099, 0.255 |

BID, twice daily. CI, confidence interval. FEV_1_, forced expiratory volume in 1 second. ITT, intent-to-treat. LS, least squares. SD, standard deviation.
SE, standard error.

**Table S4** Change from baseline in trough FEV_1_ on Day 7, GOLDEN 6 (efficacy population)

| **Parameter** | **Placebo** | **Glycopyrrolate** | | | | **Aclidinium** |
| --- | --- | --- | --- | --- | --- | --- |
|  | **(*n* = 92)** | **3 µg BID**  **(*n* = 91)** | **6.25 µg BID**  **(*n* = 92)** | **12.5 µg BID**  **(*n* = 90)** | **50 µg BID**  **(*n* = 92)** | **400 µg BID**  **(*n* = 94)** |
| **Baseline FEV_1_** | | | | | | |
| *n* | 92 | 91 | 92 | 90 | 92 | 94 |
| Mean (SD), L | 1.374 (0.427) | 1.363 (0.429) | 1.380 (0.440) | 1.347 (0.408) | 1.370 (0.418) | 1.395 (0.464) |
| **Change from baseline in FEV_1_ on Day 7** | | | | | | |
| *n* | 86 | 86 | 88 | 86 | 86 | 86 |
| Mean (SD), L | –0.034 (0.251) | –0.012 (0.186) | 0.063 (0.200) | 0.097 (0.211) | 0.101 (0.220) | 0.120 (0.187) |
| LS mean (SE), L | –0.028 (0.030) | –0.016 (0.030) | 0.054 (0.030) | 0.081 (0.030) | 0.109 (0.030) | 0.129 (0.030) |
| 95% CI | –0.088, 0.032 | –0.076, 0.044 | –0.006, 0.114 | 0.021, 0.141 | 0.049, 0.169 | 0.068, 0.189 |
| **Placebo-adjusted change from baseline in FEV_1_ on Day 7** | | | | | | |
| LS mean (SE), L | - | 0.013 (0.023) | 0.082 (0.023) | 0.109 (0.023) | 0.138 (0.023) | 0.157 (0.023) |
| 95% CI | - | –0.032, 0.057 | 0.038, 0.126 | 0.064, 0.153 | 0.093, 0.182 | 0.112, 0.201 |

BID, twice daily. CI, confidence interval. FEV_1_, forced expiratory volume in 1 second. LS, least squares. SD, standard deviation. SE, standard error.

**Table S5** Standardized change from baseline in FEV_1_ AUC_0–12_ on Day 7 and Day 28, GOLDEN 2 (ITT population)

| **Parameter** | **Placebo** | **Glycopyrrolate** | | | |
| --- | --- | --- | --- | --- | --- |
|  | **(*n* = 57)** | **12.5 µg BID**  **(*n* = 55)** | **25 µg BID**  **(*n* = 54)** | **50 µg BID**  **(*n* = 57)** | **100 µg BID**  **(*n* = 59)** |
| **Baseline FEV_1_** | | | | | |
| *n* | 57 | 54 | 54 | 57 | 59 |
| Mean (SD), L | 1.169 (0.403) | 1.227 (0.434) | 1.205 (0.425) | 1.243 (0.449) | 1.202 (0.463) |
| **Standardized change from baseline in FEV_1_ AUC_0–12_ on Day 7** | | | | | |
| *n* | 54 | 54 | 53 | 54 | 59 |
| Mean (SD), L | 0.003 (0.129) | 0.142 (0.168) | 0.167 (0.200) | 0.153 (0.185) | 0.210 (0.143) |
| LS mean (SE), L | –0.003 (0.023) | 0.140 (0.023) | 0.162 (0.024) | 0.150 (0.023) | 0.207 (0.022) |
| 95% CI | –0.049, 0.043 | 0.094, 0.186 | 0.116, 0.209 | 0.104, 0.195 | 0.163, 0.251 |
| **Placebo-adjusted change from baseline in FEV_1_ AUC_0–12_ on Day 7** | | | | | |
| LS mean (SE), L | - | 0.143 (0.033) | 0.165 (0.033) | 0.153 (0.032) | 0.210 (0.032) |
| 95% CI | - | 0.079, 0.207 | 0.101, 0.230 | 0.089, 0.216 | 0.147, 0.273 |
| **Standardized change from baseline in FEV_1_ AUC_0–12_ on Day 28** | | | | | |
| *n* | 53 | 51 | 49 | 54 | 57 |
| Mean (SD), L | 0.006 (0.154) | 0.137 (0.171) | 0.172 (0.247) | 0.107 (0.205) | 0.182 (0.185) |
| LS mean (SE), L | –0.000 (0.023) | 0.135 (0.024) | 0.163 (0.024) | 0.105 (0.023) | 0.183 (0.023) |
| 95% CI | –0.046, 0.046 | 0.089, 0.182 | 0.116, 0.210 | 0.060, 0.151 | 0.138, 0.227 |
| **Placebo-adjusted change from baseline in FEV_1_ AUC_0–12_ on Day 28** | | | | | |
| LS mean (SE), L | - | 0.136 (0.033) | 0.163 (0.033) | 0.105 (0.033) | 0.183 (0.032) |
| 95% CI | - | 0.071, 0.201 | 0.098, 0.228 | 0.042, 0.169 | 0.120, 0.246 |

AUC, area under the curve. BID, twice daily. CI, confidence interval. FEV_1_, forced expiratory volume in 1 second. ITT, intent-to-treat. LS, least squares.
SD, standard deviation. SE, standard error.

**Table S6** Standardized change from baseline in FEV_1_ AUC_0–12_ on Day 7, GOLDEN 6 (efficacy population)

| **Parameter** | **Placebo** | **Glycopyrrolate** | | | | **Aclidinium** |
| --- | --- | --- | --- | --- | --- | --- |
|  | **(*n* = 92)** | **3 µg BID**  **(*n* = 91)** | **6.25 µg BID**  **(*n* = 92)** | **12.5 µg BID**  **(*n* = 90)** | **50 µg BID**  **(*n* = 92)** | **400 µg BID**  **(*n* = 94)** |
| **Baseline FEV_1_** | | | | | | |
| *n* | 92 | 91 | 92 | 90 | 92 | 94 |
| Mean (SD), L | 1.374 (0.427) | 1.363 (0.429) | 1.380 (0.440) | 1.347 (0.408) | 1.370 (0.418) | 1.395 (0.464) |
| **Standardized change from baseline in FEV_1_ AUC_0–12_ on Day 7** | | | | | | |
| *n* | 92 | 91 | 92 | 89 | 92 | 90 |
| Mean (SD), L | –0.026 (0.214) | 0.034 (0.178) | 0.068 (0.181) | 0.122 (0.178) | 0.169 (0.211) | 0.153 (0.224) |
| LS mean (SE), L | –0.020 (0.032) | 0.032 (0.032) | 0.064 (0.032) | 0.105 (0.032) | 0.176 (0.032) | 0.170 (0.032) |
| 95% CI | –0.083, 0.043 | –0.031, 0.095 | 0.001, 0.127 | 0.042, 0.168 | 0.113, 0.239 | 0.107, 0.233 |
| **Placebo-adjusted change from baseline in FEV_1_ AUC_0–12_ on Day 7** | | | | | | |
| LS mean (SE), L | - | 0.053 (0.018) | 0.084 (0.019) | 0.126 (0.019) | 0.196 (0.018) | 0.190 (0.019) |
| 95% CI | - | 0.016, 0.089 | 0.048, 0.121 | 0.089, 0.162 | 0.160, 0.233 | 0.154, 0.227 |

AUC, area under the curve. BID, twice daily. CI, confidence interval. FEV_1_, forced expiratory volume in 1 second. LS, least squares. SD, standard deviation.
SE, standard error.

**Table S7** Change from baseline in peak FEV_1_ on Day 28, GOLDEN 2 (ITT population)

| **Parameter** | **Placebo** | **Glycopyrrolate** | | | |
| --- | --- | --- | --- | --- | --- |
|  | **(*n* = 57)** | **12.5 µg BID**  **(*n* = 55)** | **25 µg BID**  **(*n* = 54)** | **50 µg BID**  **(*n* = 57)** | **100 µg BID**  **(*n* = 59)** |
| **Baseline FEV_1_** | | | | | |
| *n* | 57 | 54 | 54 | 57 | 59 |
| Mean (SD), L | 1.169 (0.403) | 1.227 (0.434) | 1.205 (0.425) | 1.243 (0.449) | 1.202 (0.463) |
| **Change from baseline in peak FEV_1_ on Day 28** | | | | | |
| *n* | 53 | 51 | 49 | 53 | 57 |
| Mean (SD), L | 0.093 (0.178) | 0.261 (0.188) | 0.296 (0.263) | 0.255 (0.224) | 0.317 (0.214) |
| LS mean (SE), L | 0.086 (0.029) | 0.254 (0.029) | 0.285 (0.030) | 0.251 (0.029) | 0.318 (0.028) |
| 95% CI | 0.029, 0.143 | 0.196, 0.312 | 0.226, 0.343 | 0.194, 0.307 | 0.263, 0.373 |
| **Placebo-adjusted change from baseline in peak FEV_1_ on Day 28** | | | | | |
| LS mean (SE), L | - | 0.168 (0.041) | 0.199 (0.041) | 0.165 (0.041) | 0.232 (0.040) |
| 95% CI | - | 0.087, 0.249 | 0.117, 0.280 | 0.085, 0.245 | 0.153, 0.311 |

BID, twice daily. CI, confidence interval. FEV_1_, forced expiratory volume in 1 second. ITT, intent-to-treat. LS, least squares. SD, standard deviation.
SE, standard error.

**Figure S1** Least squares mean change from baseline in FEV_1_ over time on Day 28 (GOLDEN 2 Substudy^a^ ITT population)


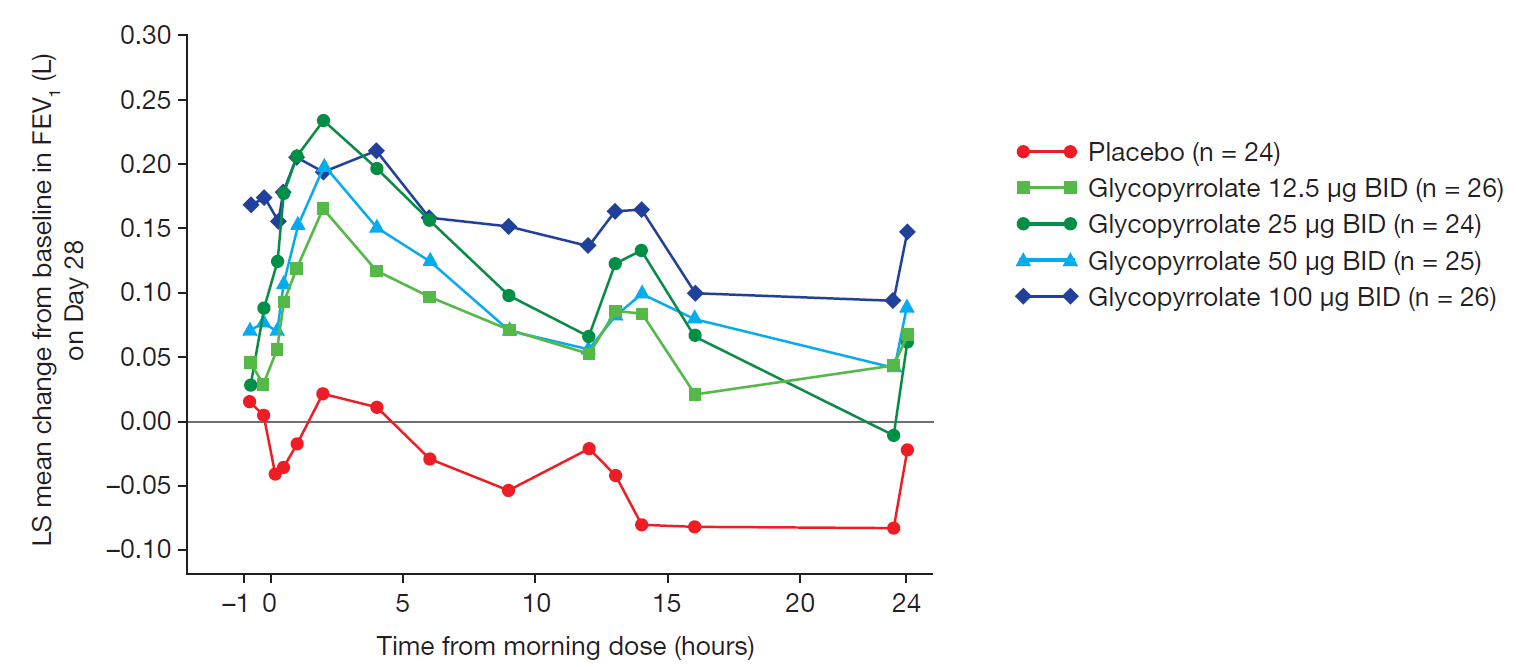


^a^Measurement of FEV_1_ over time was performed in a subset of subjects.

BID, twice daily. FEV_1_, forced expiratory volume in 1 second. ITT, intent-to-treat. LS, least squares.

**Figure S2** Mean change from baseline in FEV_1_ over time on Day 7 (GOLDEN 6 efficacy population)


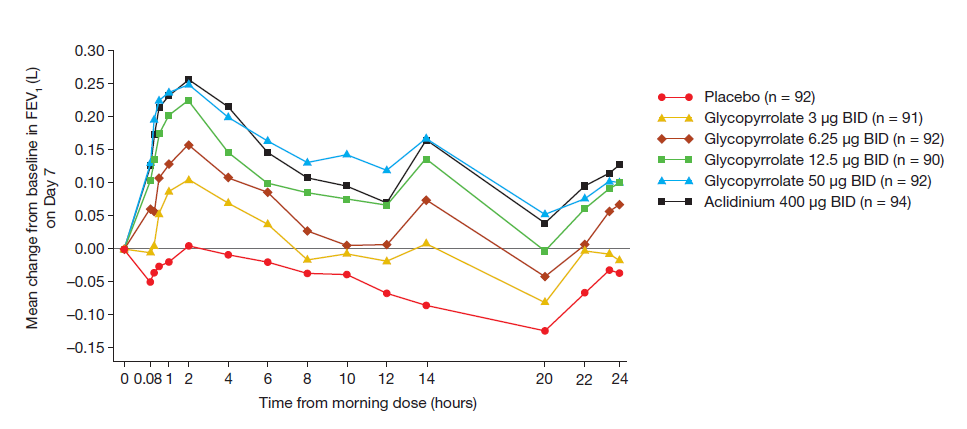


BID, twice daily. FEV_1_, forced expiratory volume in 1 second.
